# Virtual Reality–Based Assessment of Attention-Deficit/Hyperactivity Disorder and Comorbid Symptoms in Children: Framework Development and Standardization Study

**DOI:** 10.2196/69146

**Published:** 2025-10-07

**Authors:** Harim Jeong, Minjoo Kang, Kennet Sorenson, Jacob Moore, Robert James Blair, Ellen Leibenluft, Jeffrey H Newcorn, Beth Krone, Singi Jeong, Donghee Kim, Soonjo Hwang

**Affiliations:** 1 Department of Psychiatry University of Nebraska Medical Center Omaha, NE United States; 2 University of Nebraska Medical Center Omaha, NE United States; 3 Mental Health Services in the Capital Region of Denmark Brondby Denmark; 4 National Institute of Mental Health Bethesada, MD United States; 5 Icahn School of Medicine at Mount Sinai New York, NY United States; 6 Department of Electrical and Computer Engineering Sungkyunkwan University Seoul Republic of Korea; 7 Department of Computer Science and Engineering Sungkyunkwan University Seoul Republic of Korea

**Keywords:** virtual reality, attention-deficit/hyperactivity disorder, ADHD, behavioral assessment, digital health

## Abstract

**Background:**

As virtual reality (VR) technology becomes increasingly prevalent, its potential for collecting objective behavioral data in psychiatric settings has been widely recognized. However, the lack of standardized methodologies limits reproducibility and data integration across studies, particularly in assessing attention-deficit/hyperactivity disorder (ADHD) and associated behaviors, such as irritability and aggression.

**Objective:**

This study examines the use of VR-based movement data to operationalize core ADHD symptoms (hyperactivity and inattention) and comorbid disruptive behaviors (irritability and aggression), aiming to identify reproducible and clinically actionable metrics and evaluate their explanatory power for each symptom domain to assess the overall use of these variables.

**Methods:**

A total of 45 children (mean age 9.06, SD 2.11 years; n=14/45, 31% female) participated in the study and were divided into 2 groups: 28 (62%) diagnosed with ADHD and 17 (38%) controls. Seven VR-derived movement variables were analyzed: average speed, acceleration, total distance, area occupied, distance between the hands and head, frequency of movement, and time spent still. Correlation and stepwise regression analyses identified which variables best predicted ADHD symptoms and comorbid behaviors.

**Results:**

Among the 7 VR-derived variables, average speed (mean *r*=0.460, SD 0.097) and total distance (mean *r*=0.442, SD 0.116) showed the broadest associations, each correlating with 8 measures. In contrast, frequency of movement was related only to hyperactivity (*r*=0.416; *P*=.004), suggesting strong but narrow predictive value. Stepwise regression identified total distance as the sole and strongest predictor of hyperactivity (*R*^2^=0.411) and, except for participant-reported irritability, yielded significant models for all other measures (mean *R*^2^=0.282, SD 0.064; all *P*<.05).

**Conclusions:**

This study provides empirical evidence on VR-derived movement variables that can inform the development of standardized methodologies for ADHD and comorbid behavior assessment. The identified metrics and their predictive patterns offer a basis for integrating VR-based measures into future research and clinical applications.

## Introduction

### Background

The application of virtual reality (VR) technology in psychiatry holds significant promise for enhancing diagnostic accuracy and complementing traditional assessment methods, particularly in disorders such as attention-deficit/hyperactivity disorder (ADHD) and its comorbid symptoms, including irritability and aggression [1-4]. Traditional psychiatric assessments often rely on subjective self-reports and parent reports, which can be influenced by recall and other biases and can be limited to clinical settings that may not reflect a patient’s behavior in real-world environments [5,6]. These challenges are particularly pronounced in youth populations, where self-reports may be inconsistent, parent reports may not fully capture key psychopathologies, and discrepancies between multiple informants are common [7,8]. VR addresses these limitations by enabling controlled, realistic simulations in which behavior can be objectively observed and recorded, offering a detailed view of symptom manifestation in various settings that are more representative of daily life [9].

Specifically, VR’s potential is particularly relevant for psychiatric disorders in youth with ADHD and its behavioral symptoms, as it allows for the collection of precise behavioral data—including attention span, impulsivity, and hyperactivity—which is critical for understanding the symptom manifestations in naturalistic settings [10]. Given that these symptoms often vary with context and observer attributes, VR provides a unique advantage by enabling data collection in controlled, dynamic environments that mimic real-life scenarios, thereby reducing the need to rely solely on subjective reporting by patients or caregivers [11]. This positions VR as a promising complementary assessment tool to traditional assessments.

Despite the promise VR holds in ADHD, practical implementation in a clinical setting faces several challenges. Most existing studies have small sample sizes and lack standardized frameworks for defining how specific types of VR-collected data relate to ADHD and comorbid disruptive behavior symptoms. This lack of standardization creates challenges for developing generalized VR assessment tools that can be reliably replicated and validated across diverse populations [12,13]. This gap highlights the need for systematic research that clarifies the roles of individual VR-derived data elements in the assessment of ADHD and comorbid disruptive behavior symptoms, ultimately facilitating VR’s integration into clinical practice as an objective, data-driven tool.

### Objectives

To address these challenges, this study seeks to define and evaluate specific behavioral data collected through VR that are essential for assessing ADHD and comorbid disruptive behavior symptoms. By establishing a clear mapping between VR data types (eg, attention-related behaviors and impulsivity indicators) and ADHD or disruptive behavior symptom domains, this study aims to create a replicable framework that other researchers and clinicians can use. Our goal is to provide a standardized framework that can facilitate consistent data collection across studies and enable the accumulation of larger sample data over time to build the evidence base required to eventually apply VR-based tools in clinical settings for ADHD and comorbid disruptive behavior symptom assessment.

Therefore, this study is guided by the following research questions (RQs):

1. Which specific types of VR-derived behavioral data are most relevant in assessing ADHD and comorbid disruptive behavior symptoms?
2. How can these VR-derived data elements be combined into a composite measure to enhance the comprehensive detection of ADHD and comorbid disruptive behavior symptoms, thereby contributing to the development of a standardized assessment tool?

Through this research, we aim to lay a foundational framework that can be built upon in future studies, thereby facilitating the clinical adoption of VR-based ADHD and comorbid disruptive behavior symptom assessment tools. By providing a clear and replicable structure for VR data collection and interpretation, this study seeks to support the accumulation of evidence necessary for integrating VRs into clinical settings and disorders, ultimately contributing to more accessible, objective, and accurate assessments in clinical settings.

## Methods

### Experimental Setting

The study recruited 45 children (mean age 9.06, SD 2.11 years), including 14 (31%) girls. Participants were divided into 2 groups: 28 (62%) children diagnosed with ADHD and 17 (38%) children with no history of ADHD or related psychiatric diagnoses. This sample composition allowed us to capture diverse behavioral patterns and identify ADHD and comorbid disruptive behavior symptom–specific movement characteristics within the VR environment. Rather than dividing the ADHD and control groups in our analyses, we focused on the continuum of movement data across both groups, aligning with the research domain criteria framework, which emphasizes transdiagnostic dimensions of behavior and neurobiology rather than categorical diagnoses. This approach allowed us to examine how specific movement patterns may reflect core symptom dimensions across individuals, regardless of diagnostic labels.

**Figure 1:**
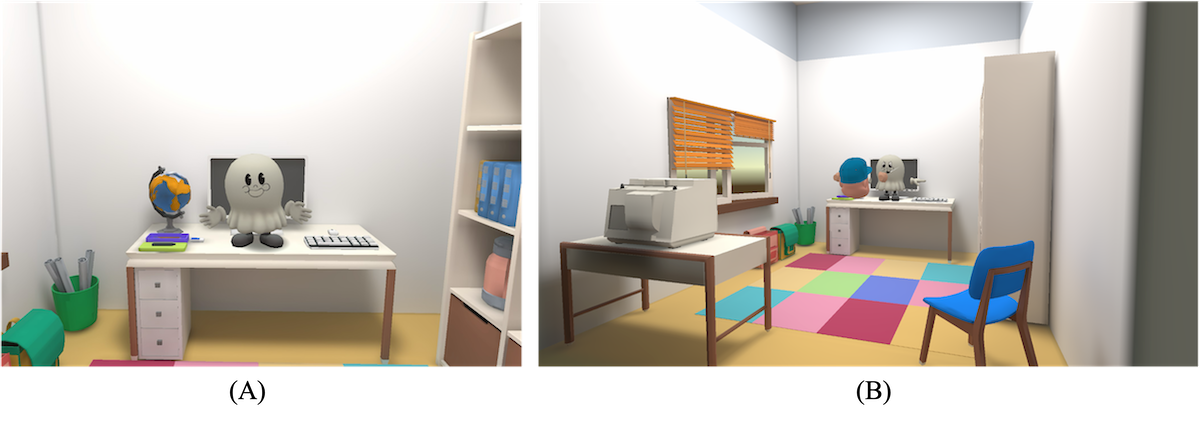
Virtual environment used to assess attention-deficit/hyperactivity disorder and comorbid behaviors in children through naturalistic interaction during a virtual reality session. (A) Participant’s first-person view; (B) overview of the virtual room layout.

A 5-minute VR session, developed using Meta’s Oculus Quest 2 system and Unity (Unity Technologies), was used as the experimental setting. Within this session, children engaged in unstructured interactions, as no specific instructions or goals were provided. Instead, a virtual avatar initiated casual conversation prompts (eg, asking about the participant’s day or mood), allowing for spontaneous verbal and nonverbal response. The avatar also responded to physical actions, such as getting closer to the avatar (proximity) or being struck by the participant, allowing a broader range of naturalistic interaction. The virtual room setting was designed to mimic a real-life environment with familiar objects (eg, desk, chair, and bookshelf) spatially arranged. This setup aimed to evoke naturalistic behaviors, such as exploring the space, interacting with objects in the room, and engaging in casual conversations with the virtual avatar about daily routines and moods. Figure 1 provides an illustration of the VR environment.

Movement data were recorded as 3D coordinates (x, y, and z) at 0.5-second intervals using the head-mounted display and hand controllers. These data reflected real-time behavior, including locomotion, gesture, and object-directed movement, and were continuously monitored throughout the session. By structuring the VR interaction into a natural, task-free conversational format, we aimed to gather unbiased behavioral data reflecting the children’s spontaneous movements and social interaction styles to specifically capture movement patterns associated with core ADHD symptoms (eg, hyperactivity and inattention) and comorbid disruptive behavior symptoms (eg, irritability and aggression).

### Data Processing

The recorded 3D coordinates of participants’ head and hand movements were subsequently processed to derive behavioral indicators for analysis. These variables were then categorized into 3 main groups.

The first group of data includes velocity-related features, including the average speed of head and hand movements and acceleration, which is defined as the rate of change of speed over time for the head and hands. These variables were chosen based on evidence suggesting that children with ADHD tend to exhibit faster movements and more frequent acceleration than those without the condition [14,15].

The second group of data was spatial features, which include the total distance moved by the head and hands, the area occupied by a participant during movement, and the distance between the hands and head. These variables were selected based on research indicating that body movement can vary significantly across different psychiatric disorders [16]. Specifically, the distance between the hands and head is a movement pattern associated with certain mental health conditions, such as ADHD [17].

The final group of data was frequency and duration features, which identify how often a participant moves and measure the time the participant spends not moving. These variables were selected because frequent movement or prolonged stillness can indicate manifestations of hyperactivity symptoms in children with ADHD [18].

In total, 7 variables (average speed, acceleration, total distance, area occupied, distance between hands and head, frequency of movement, and time spent still) representing these 3 distinct characteristics were derived from the raw data for analysis. The selected variables and their calculation methods are detailed in Table 1.

**Table 1:** Definitions and equations for the 7 virtual reality (VR)–derived movement variables used to assess attention-deficit/hyperactivity disorder and comorbid disruptive behaviors in children during a VR session.

Category and variables			Equations		References	
**Velocity**						[14,19-21]
	Average speed	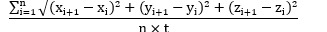				
	Acceleration	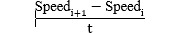				
**Spatial**						[16,22-24]
	Total distance	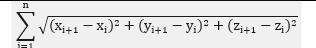				
	Area occupied	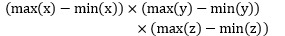				
	Distance between the hands and head	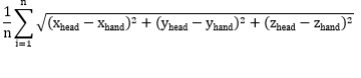				
**Frequency and duration**						[25-27]
	Frequency of movement	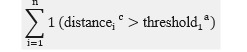				
	Time spent still	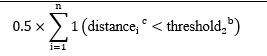				

^a^The “threshold_1_” is the minimum distance for detecting movement.

^b^The “threshold_2_” represents the maximum distance within which a participant is considered stationary.

^c^The “distance” refers to the Euclidean distance calculated between consecutive positions of the head or hands.

### External Psychopathology Assessment

First, the ADHD symptom profile was evaluated with the ADHD Rating Scale (ADHD-RS) [28]. The ADHD-RS is an 18-item questionnaire scored on a 0 to 3 scale, with 9 questions related to hyperactivity and 9 related to inattention, completed by parents [29]. The ADHD-RS was used to assess the core symptoms of ADHD—hyperactivity and inattention—by examining their relationship with movement features captured through VR.

To assess participants’ disruptive behavior symptoms, we collected relevant data using validated assessment tools. In addition to ADHD core symptoms, the disruptive behavior symptoms evaluated in this study included irritability and reactive and proactive aggression. Irritability was included as it is one of the most common features related to various psychopathologies in youth, including ADHD [30]. Thus, we hypothesized that irritability could be predicted through movement analysis in a virtual environment. Data on irritability were collected from both parents and participants using the Affective Reactivity Index (ARI), a 7-item questionnaire scored on a 0 to 2 scale [31].

Next, we assessed reactive and proactive aggression using the Reactive-Proactive Aggression Questionnaire (RPQ) [32]. Aggressive cognitions are more likely to become aggressive actions in youth with ADHD and impulse control. Thus, aggressive behavior can be a relevant target for prediction through movement data in virtual settings [2]. To leverage VR’s capability to capture and analyze these behaviors in a controlled environment, we included the RPQ in our study. The RPQ consists of 23 items, each scored on a 0 to 2 scale, with data collected from both parents and participants. The specific items for each assessment tool used in this study are provided in Multimedia Appendix 1.

### Analysis of Psychopathology Descriptive Statistics

To explore the relationship between VR-derived movement data and ADHD or disruptive behavior symptoms, we first calculated descriptive statistics for all psychopathology measures. This step provided an overview of the data distribution and informed the subsequent analyses, and these scores were used as dependent variables in the analysis.

### Analysis to Identify Key VR-Derived Predictors of ADHD and Comorbid Symptoms

We then conducted Pearson correlation analyses to examine associations between VR-derived movement variables and assessment scores. This step helped determine which specific behavioral indicators captured in the VR environment were most relevant to ADHD and comorbid disruptive behavior symptoms.

### Analysis to Identify Core ADHD Symptoms Captured by VR-Derived Data

We applied a backward stepwise multiple linear regression (entry criterion: *P*<.05; removal criterion: *P*>.10) to identify the most predictive combination of VR-derived variables for each symptom domain. Multicollinearity among predictors was assessed after model selection using the variance inflation factor (VIF), with VIF<5 considered acceptable.

### Ethical Considerations

Participants were recruited from a university hospital and the local community in the central United States. Written informed consent was obtained from both the children and their legal guardians before participation. The study protocol was approved by the institutional review board at the University of Nebraska Medical Center (580-23). All collected data were deidentified to ensure confidentiality and privacy. Participants received no compensation for their involvement in the study. No identifiable images of participants were collected or included in the manuscript.

## Results

### Descriptive Statistics of Psychopathology Measures

The ADHD-RS scores had a mean score of 10.44 (SD 7.24) for hyperactivity, 11.8 (SD 7.33) for inattention, and 22.24 (SD 13.23) for the total ADHD-RS score. The ARI scores, collected from both parents and participants, showed mean scores of 4.31 (SD 3.42) for parent-reported data and 4.35 (SD 3.55) for participant-reported data. For reactive and proactive aggression, as measured by the RPQ, the parent-reported reactive aggression had a mean of 9.15 (SD 5.29), proactive aggression had a mean of 2.37 (SD 3.29), and the total RPQ score had a mean of 11.84 (SD 8.44). Participant-reported RPQ scores had mean scores of 7.6 (SD 4.38) for reactive aggression and 2.11 (SD 2.42) for proactive aggression and a total mean score of 10.04 (SD 6.39).

These detailed descriptive statistics offer foundational insights into the distribution of each dependent variable and are further analyzed in the subsequent sections. Full descriptive statistics are presented in Table 2.

**Table 2:** Descriptive statistics of attention-deficit/hyperactivity disorder (ADHD) symptoms and comorbid disruptive behavior scores (ADHD Rating Scale [ADHD-RS], Affective Reactivity Index [ARI], and Reactive-Proactive Aggression Questionnaire [RPQ]) reported by parents and participants.

Assessment tool and score type			Scores, mean (SD)
**ADHD-RS**			
	Hyperactivity	10.44 (7.24)	
	Inattention	11.8 (7.33)	
	Total	22.24 (13.23)	
ARI total (parent)			4.31 (3.42)
ARI total (participant)			4.35 (3.55)
**RPQ (parent)**			
	Reactive	9.15 (5.29)	
	Proactive	2.37 (3.29)	
	Total	11.84 (8.44)	
**RPQ (participant)**			
	Reactive	7.6 (4.38)	
	Proactive	2.11 (2.42)	
	Total	10.04 (6.39)	

### Identifying Key VR-Derived Predictors of Core ADHD Symptoms and Comorbid Disruptive Behavior Symptoms

Several VR-derived movement variables showed significant correlations with ADHD and comorbid symptom scores, indicating specific behavioral patterns associated with these conditions. Among the velocity-related variables, average speed showed strong correlations with 8 assessment scores, including the ADHD-RS hyperactivity (*r*=0.639; *P*<.001) and inattention (*r*=0.500; *P*<.001) subscales; the parent-reported RPQ total score (*r*=0.504; *P*<.001), proactive aggression subscore (*r*=0.477; *P*<.001), and reactive aggression subscore (*r*=0.464; *P*=.001); and youth-reported RPQ total score (*r*=0.394; *P*=.007), proactive subscore (*r*=0.367; *P*=.01), and reactive subscore (*r*=0.332; *P*=.03).

Regarding the spatial variables, the area occupied was significantly correlated with 5 assessment scores, including the participant-reported RPQ proactive aggression subscore (*r*=0.362; *P*=.01) and the ADHD-RS hyperactivity subscale (*r*=0.357; *P*=.02), the youth-reported RPQ total score (*r*=0.327; *P*=.03) and the parent-reported RPQ reactive aggression subscore (*r*=0.314; *P*=.03), and the ADHD-RS inattention subscore (*r*=0.295; *P*=.049). Furthermore, the total distance variable was significantly correlated with 8 assessment scores, including the ADHD-RS hyperactivity (*r*=0.641; *P*<.001) and inattention (*r*=0.494; *P*<.001) subscales; the parent-reported RPQ total scores (*r*=0.498; *P*<.001), proactive aggression subscore (*r*=0.503; *P*<.001), and reactive aggression subscore (*r*=0.439; *P*=.002); the youth-reported RPQ proactive (*r*=0.323; *P*=.03) and total (*r*=0.302; *P*=.04); and the parent-reported ARI score (*r*=0.337; *P*=.02).

Finally, in the category of frequency and duration variables, the frequency of movement showed a significant correlation with the ADHD-RS hyperactivity subscale (*r*=0.416; *P*=.004). The time spent still variable was negatively correlated with 6 assessment scores, including the ADHD-RS hyperactivity (*r*=−0.448; *P*=.002) and inattention (*r*=−0.363; *P*=.01) subscales, the parent-reported RPQ reactive (*r*=−0.349; *P*=.02) and total (*r*=−0.349; *P*=.02) scores, and the participant-reported RPQ reactive (*r*=−0.317; *P*=.03) and total (*r*=−0.333; *P*=.03) scores. These correlations are summarized in Table 3.

**Table 3:** Significant correlations between virtual reality (VR)–derived movement variables and attention-deficit/hyperactivity disorder (ADHD) and comorbid symptom scores (ADHD Rating Scale [ADHD-RS], Affective Reactivity Index [ARI], and Reactive-Proactive Aggression Questionnaire [RPQ]) among children participating in a VR-based behavioral assessment study.

VR data element and assessment score		Pearson correlation coefficient (*r*)	*P* value
**Average speed**			
	ADHD-RS hyperactivity	0.639	<.001
	ADHD-RS inattention	0.500	<.001
	RPQ parent total	0.504	<.001
	RPQ parent proactive	0.477	<.001
	RPQ parent reactive	0.464	.001
	RPQ participant total	0.394	.007
	RPQ participant proactive	0.367	.01
	RPQ participant reactive	0.332	.03
**Area occupied**			
	ADHD-RS hyperactivity	0.357	.02
	ADHD-RS inattention	0.295	.05
	RPQ participant total	0.327	.03
	RPQ participant proactive	0.362	.01
	RPQ parent reactive	0.314	.04
**Total distance**			
	ADHD-RS hyperactivity	0.641	<.001
	ADHD-RS inattention	0.494	<.001
	RPQ parent total	0.498	<.001
	RPQ parent proactive	0.503	<.001
	RPQ parent reactive	0.439	.002
	RPQ participant proactive	0.323	.03
	RPQ participant total	0.302	.04
	ARI parent	0.337	.02
**Frequency of movement**			
	ADHD-RS hyperactivity	0.416	.004
**Time spent still**			
	ADHD-RS hyperactivity	−0.448	.002
	ADHD-RS inattention	−0.363	.01
	RPQ parent reactive	−0.349	.02
	RPQ parent total	−0.349	.02
	RPQ participant reactive	−0.317	.03
	RPQ participant total	−0.333	.03

### Identifying Core ADHD Symptoms and Comorbid Disruptive Behavior Symptoms Captured by VR-Derived Data

Stepwise regression identified specific combinations of VR-derived movement variables that best predicted ADHD and comorbid symptom scores. The most influential predictors varied by symptom profile, highlighting the ability of VR-based behavioral data to differentiate psychological characteristics.

As all candidate variables represent aspects of physical movement, variables such as total distance (VIF=13.05) and average speed (VIF=13.17) exhibited high VIF values during preliminary examination before final model selection. However, due to the nature of the stepwise regression procedure, only one of these correlated predictors was selected in each final model. As assessed after model selection, all variables retained in the final models showed acceptable VIF levels (<5), indicating that multicollinearity was not a concern for interpretation.

For the parent-reported ARI score related to irritability, the final model identified 2 predictors: total distance (β=.1327; *P*<.001) and frequency of movement (β=−.0807; *P*=.003), with an *R*^2^ of 0.287. The positive association with total distance indicates that greater movement distance is linked to higher irritability, whereas the negative association with movement frequency suggests that more frequent movements are linked to lower irritability.

For the parent-reported RPQ total score, 2 predictors remained in the final model: average speed (β=25.6122; *P*<.001) and frequency of movement (β=−.1270; *P*=.03), with an *R*^2^ of 0.334. The positive association with average speed indicates that faster movements are related to higher total aggression scores, while the negative association with frequency of movement suggests that frequent movements are associated with lower aggression.

In the parent-reported RPQ proactive aggression score, only total distance (β=.0793; *P*<.001) was retained, with an *R*^2^ of 0.253. This suggests that proactive aggression is primarily captured by the overall distance covered in the VR environment. For the parent-reported RPQ reactive aggression score, 2 variables were significant: average speed (β=15.8999; *P*<.001) and frequency of movement (β=−.0886; *P*=.02), with an *R*^2^ of 0.313. The results show that faster movements are associated with higher reactive aggression, while frequent movements correlate negatively with reactive aggression.

In the youth-reported RPQ scores, the total aggression score was best explained by average speed (β=17.8567; *P*<.001) and frequency of movement (β=−.1119; *P*=.02), with an *R*^2^ of 0.263. For proactive aggression, the model included average speed (β=6.3336; *P*=.001) and frequency of movement (β=−.0399; *P*=.03), with an *R*^2^ of 0.231. The reactive aggression score was also predicted by average speed (β=10.5277; *P*=.003) and frequency of movement (β=−.0678; *P*=.04), with an *R*^2^ of 0.194. These results consistently show that average speed and frequency of movement predicted youth-reported aggression scores.

For the ADHD-RS scores, the hyperactivity subscale was significantly explained by total distance (β=.2219; *P*<.001), with an *R*^2^ of 0.411, indicating that hyperactivity is explained by the overall distance covered in the VR tasks. The inattention subscale was best predicted by average speed (β=14.0459; *P*<.001), with an *R*^2^ of 0.250, showing that faster movements are linked to higher inattention scores. The detailed results supporting these observations are provided in Table 4.

**Table 4:** Stepwise regression models identifying virtual reality (VR)–derived movement predictors of attention-deficit/hyperactivity disorder (ADHD) symptoms and comorbid symptom scores (ADHD Rating Scale [ADHD-RS], Affective Reactivity Index [ARI], and Reactive-Proactive Aggression Questionnaire [RPQ]) in children participating in a VR-based behavioral assessment study.

Assessment score and predictor		Coefficient (β)	*P* value	*R* ^2^	
**ADHD-RS hyperactivity**					
	Total distance	0.2219	<.001	0.411	
**ADHD-RS inattention**					
	Average speed	14.0459	<.001	0.250	
**ARI (parent)**					0.287
	Total distance	0.1327	<.001		
	Frequency of movement	−0.0807	.003		
ARI (participant)		—^a^	—	—	
**RPQ total (parent)**					0.334
	Average speed	25.6122	<.001		
	Frequency of movement	−0.1270	.03		
**RPQ proactive (parent)**					
	Total distance	0.0793	<.001	0.253	
**RPQ reactive (parent)**					0.313
	Average speed	15.8999	<.001		
	Frequency of movement	−0.0886	.02		
**RPQ total (participant)**					0.263
	Average speed	17.8567	<.001		
	Frequency of movement	−0.1119	.02		
**RPQ proactive (participant)**					0.231
	Average speed	6.3336	.001		
	Frequency of movement	−0.0399	.03		
**RPQ reactive (participant)**					0.194
	Average speed	10.5277	.003		
	Frequency of movement	−0.0678	.04		
^a^indicates that no variables were statistically significant for this model.					

## Discussion

### Principal Findings

This study explored the potential of VR-collected movement data to predict ADHD and comorbid disruptive behavior symptoms, aiming to bridge the gap between theoretical possibilities and practical clinical applications. Specifically, we sought to identify VR-derived movement data elements that effectively capture symptoms of ADHD or comorbid disruptive behavior and to examine how these elements can contribute to reliable predictive models for behavioral symptoms.

Our findings suggest that VR-derived data can play a significant role in capturing symptoms of ADHD or disruptive behavior, offering insights that may improve the accuracy, objectivity, and accessibility of assessment tools [33]. By establishing foundational evidence on the associations between VR data and behavioral symptoms, this study may contribute to the broader goal of incorporating VR into psychiatric practice to enhance care precision and accessibility for patients with externalizing psychopathologies [34].

For RQ 1, the results indicate that certain VR-derived movement variables, particularly average speed and total distance, are more closely related to ADHD and disruptive behavior symptoms than other VR-derived variables. Average speed demonstrated significant correlations with a variety of symptoms, suggesting it could function as a versatile marker of ADHD and comorbid disruptive behavior symptoms, including inattention and aggression. Meanwhile, total distance was particularly relevant for predicting hyperactivity symptoms, as shown by its strong correlation with the ADHD-RS hyperactivity subscale.

In addressing RQ 2, we explored how VR-derived behavioral features could be combined to predict specific symptom domains using stepwise regression. This approach identified the most explanatory feature combinations for each behavioral construct. For ADHD-related symptoms, hyperactivity and inattention were best predicted by total distance and average speed, respectively, indicating relatively direct behavioral correlates for each symptom. In contrast, symptoms related to irritability and aggression (eg, ARI and RPQ scores) required the inclusion of additional features, such as frequency of movement, to improve model fit. These findings imply that comorbid disruptive behaviors may involve more complex behavioral dynamics than core ADHD symptoms [35], possibly reflecting more nuanced or multifactorial movement patterns within the VR environment.

### Comparison With Prior Work

Although we collected both parent and child symptom ratings, our analyses showed that movement patterns recorded in VR were more strongly associated with parent reports, as reflected in consistently higher *R*^2^ values across models. This may reflect differences in how each informant perceives and interprets the behavior of parents, as externalizing psychopathologies tend to be outwardly observable better by parents (eg, hyperactivity or aggression), as children may be more attuned to internal states such as anxiety or mood [36]. As our study focused on externalizing problems, such as ADHD, irritability, and aggression, it makes sense that the behavioral signals captured in VR aligned more closely with the parent perspectives. The stronger associations with parent-reported symptoms may indicate that the VR-collected movement patterns correspond more with outwardly visible behaviors.

Furthermore, using movement-based data collected in a virtual environment may help reduce biases commonly associated with caregiver-reported assessments [37]. It offers clinicians an opportunity to observe behavioral patterns more directly and objectively. As more research accumulates using standardized, quantitatively derived behavioral features, there is potential to develop generalized benchmarks that support more efficient and timely assessment of ADHD and comorbid symptoms. This could ultimately enhance clinical workflows by complementing traditional tools with scalable, technology-assisted methods.

These findings align with previous observations that ADHD-related behaviors often manifest in increased physical activity [24], which VR technology can effectively capture in a controlled environment. The patterns observed in this study suggest that physical activity within a VR environment may reflect specific symptom dimensions. For instance, while total distance appears to align with overall physical movement associated with hyperactivity, average speed may capture movement intensity and impulsivity, characteristics often linked to symptoms such as aggression and inattention. However, it is important to note that while these associations were observed in our sample, further research is needed to determine whether these movement patterns consistently predict symptoms across diverse samples and settings.

### Limitations and Future Directions

Given the novel nature of VR-derived movement data in this study, these findings provide initial insights but also raise questions that warrant further exploration. The results point to potential indicators of symptom-specific behaviors, but additional studies with larger samples are necessary to confirm our findings and to provide further insight into the complex relations between VR variables and the symptom manifestation of various psychiatric conditions. Moreover, while average speed and total distance emerged as strong predictors for certain behaviors, the unexpected associations observed for frequency of movement and irritability highlight the complexity of interpreting VR data in relation to psychological symptoms [38].

This study is among the first to systematically examine the relationship between VR-derived movement data and ADHD or disruptive behavior symptoms in a controlled, virtual environment. These findings suggest that VR may offer a unique, real-time perspective on behavioral symptoms, providing more objective and context-rich data than traditional assessment methods.

First, the relatively small sample size and the scope of behaviors assessed may impact the generalizability of these findings. To reduce the possibility of type II error, we analyzed the entire study group without dividing them into ADHD versus healthy control groups, thereby maximizing statistical power. Nonetheless, replication with larger and more diverse samples will be necessary to confirm these results.

Second, the broad and generalized virtual environment used in this study might not fully capture the nuances specific to individual psychopathologies. While this design enabled observation of diverse behaviors, future studies should focus on developing VR scenarios tailored to specific behavioral challenges to improve the precision of VR-based assessments.

Third, the unexpected associations observed between frequency of movement and irritability highlight the complexity of interpreting VR data in relation to psychological symptoms. These findings underscore the exploratory nature of this study and the need for replication in other contexts to clarify these associations.

Future research should also consider integrating multimodal measures, such as physiological signals or neuroimaging data, to enhance interpretability and improve the predictive accuracy of VR-based assessment tools.

### Conclusions

This study responds to the growing interest in VR-based ADHD assessment by providing detailed definitions and processing methods for specific VR-derived behavioral variables, which may help improve reproducibility in future work. Our analyses identified average speed and total distance as consistent indicators across multiple symptom domains and suggested possible combinations of variables that may be particularly relevant for certain behaviors. By sharing both the design of the VR assessment and the associated data processing approach, we aim to contribute a practical reference point for other researchers. We hope that such collaborative efforts will support the continued development of VR-based tools and encourage their gradual integration into clinical assessment practices.

## Appendix

Multimedia Appendix 1 Item list for questionnaires used in the study.

## Abbreviations

ADHD: attention-deficit/hyperactivity disorder
ADHD-RS: Attention-Deficit/Hyperactivity Disorder-Rating Scale
ARI: Affective Reactivity Index
RPQ: Reactive-Proactive Aggression Questionnaire
RQ: research question
VIF: variance inflation factor
VR: virtual reality

## Data Availability

The datasets generated or analyzed during this study are available from the corresponding author on reasonable request.
