# Supplementary material for: Virtual Reality–Based Assessment of Attention-Deficit/Hyperactivity Disorder and Comorbid Symptoms in Children: Framework Development and Standardization Study
Source: JMIR Serious Games. 2025 Oct 7;13:e69146. doi: 10.2196/69146 (PMC12541263; doi:10.2196/69146)
Supplement: Multimedia Appendix 1 [file games_v13i1e69146_app1.docx]

## Appendix 1 Item List for Questionnaires Used in the Study

| Instrument | Subscale(s) | Item (short label) |
| --- | --- | --- |
| The Reactive-Proactive Aggression Questionnaire (RPQ) | Reactive | Yelled at others when annoyed |
|  |  | Reacted angrily when provoked |
|  |  | Got angry when frustrated |
|  |  | Had temper tantrums |
|  |  | Damaged things when angry |
|  |  | Angry when not getting own way |
|  |  | Angry when losing a game |
|  |  | Felt better after hitting or yelling |
|  |  | Hit others in self-defense |
|  |  | Angry or hit others when teased |
|  | Proactive | Fought to assert dominance |
|  |  | Took items from others |
|  |  | Vandalized for amusement |
|  |  | Participated in gang fight for status |
|  |  | Hurt others to win a game |
|  |  | Used force to make others comply |
|  |  | Used force to obtain money or items |
|  |  | Threatened or bullied others |
|  |  | Made obscene calls for amusement |
|  |  | Encouraged others to gang up on someone |
|  |  | Carried a weapon for fighting |
|  |  | Yelled to make others comply |
| The Affective Reactivity Index  (ARI) | Total | Easily annoyed by others |
|  |  | Often loses temper |
|  |  | Stays angry long |
|  |  | Angry most of the time |
|  |  | Gets angry often |
|  |  | Loses temper easily |
|  |  | Irritability causes problems |
| ADHD Rating Scale—5  (ADHD RS) | Inattentive | Careless mistakes in schoolwork |
|  |  | Difficulty sustaining attention |
|  |  | Does not listen when spoken to |
|  |  | Fails to follow instructions |
|  |  | Difficulty organizing tasks |
|  |  | Avoids sustained mental effort |
|  |  | Loses necessary items |
|  |  | Easily distracted by stimuli |
|  |  | Forgetful in daily activities |
|  | Hyperactivity-Impulsivity | Fidgets or squirms in seat |
|  |  | Leaves seat when expected to stay |
|  |  | Runs/climbs excessively (inappropriate) |
|  |  | Difficulty playing quietly |
|  |  | “On the go” or acts driven by motor |
|  |  | Talks excessively |
|  |  | Blurts out answers prematurely |
|  |  | Difficulty waiting turn |
|  |  | Interrupts or intrudes on others |
